# Sex differences in the blood metabolome of extremely preterm infants: a pilot study on the impact of antibiotic therapy

**DOI:** 10.1186/s13293-025-00798-1

**Published:** 2025-12-06

**Authors:** Michele Costanzo, Marianna Caterino, Sabrina Bianco, Margherita Ruoppolo, Giovanni Sotgiu, Mariangela Puci, Flavia Franconi, Ilaria Campesi

**Affiliations:** 1 Department of Molecular Medicine and Medical Biotechnology, University of Naples Federico II, Naples, 80131 Italy; 2 CEINGE – Biotecnologie Avanzate Franco Salvatore, Naples, 80145 Italy; 3 Clinical Epidemiology and Medical Statistics Unit, Department of Medicine, Surgery and Pharmacy, University of Sassari, Sassari, 07100 Italy; 4 Laboratory of Gender Pharmacology and Medicine, National Institute of Biostructures and Biosystems, Sassari, 07100 Italy; 5 Department of Biomedical Sciences, University of Sassari, Sassari, 07100 Italy

**Keywords:** Extremely preterm infants, Blood metabolomics, Antibiotics, Sex differences

## Abstract

**Background:**

Despite growing recognition of sex differences in medicine, little is known about their role in neonatology, particularly among extremely premature infants (EPI, < 28 weeks gestation), who face high morbidity and mortality driven by infections. Antibiotics therapy is widely used but may alter cellular metabolism, leading to adverse drug reactions. However, pharmacological studies in EPI remain limited, and sex-dependent effects of antibiotic treatments are largely unexplored. This study investigated sex-related metabolomic differences in EPI in relation to antibiotic exposure.

**Methods:**

Targeted mass spectrometry (MS) was applied to dried blood spots (DBS) collected within the neonatal screening program of the Campania region (Italy) between 2018 and 2023. Amino acids (AA) and acylcarnitines (AC) were quantified in 116 EPI stratified by sex and antibiotics treatment.

**Results:**

Untreated EPI of both sexes showed largely comparable metabolic profiles, with the exception of higher C16OH levels in males. Antibiotic treatment, however, markedly amplified sex-dependent divergence, with male EPI displaying significantly elevated AC concentrations (C0, C2, C3, C4, C5, C6, C5OH, C10:1, C16:1, C18, C18:1) compared to females. Stratification by penicillins + aminoglycosides treatment revealed distinct patterns: in EPI treated with a penicillins + aminoglycosides combination, males exhibited higher levels of C0, C2, C4, C6, C16:1, C18, and C18:1, while C3, C5, C5OH, and C10:1 no longer differed by sex. Furthermore, eight additional AC (C3DC, C14:1, C14, C16, C10DC, C16OH, C4OH, C16:1OH) were significantly elevated in treated males, differences that were not detected when all antibiotic classes were pooled.

**Conclusions:**

These findings demonstrate that standard empirical antibiotic therapies for prematurity exert sex-dependent effects on neonatal metabolism, with antibiotics amplifying AC alterations in males. Our results underscore the need to consider sex as a key biological variable in neonatal pharmaco-metabolomics and highlight the potential of metabolic profiling to optimize individualized treatments in EPI.

**Supplementary Information:**

The online version contains supplementary material available at 10.1186/s13293-025-00798-1.

## Abstract (articlehighlights)

Babies born extremely early, before 28 weeks of pregnancy, face many health challenges. They are very vulnerable and often need treatments like antibiotics to fight infections. In this study, we looked at tiny blood samples obtained from 116 very premature babies in the Campania region of Italy, collected between 2018 and 2023. We wanted to see if males and females responded differently to these common treatments. We found that when babies were not treated with antibiotics, males and females had very similar blood patterns. However, when antibiotics were given, males showed stronger changes in the way their bodies handled acylcarnitines, considered indirect indicators of mitochondrial function, and amino acids, which could signal a difference in how they process energy. These results show that antibiotic treatment can affect premature babies in different ways depending on whether they are males or females. This highlights the need for doctors and researchers to consider sex differences when caring for and studying premature newborns. By better understanding these differences, we can work toward safer, more personalized care for the most vulnerable babies.

**Supplementary Information:**

The online version contains supplementary material available at 10.1186/s13293-025-00798-1.

## Highlights

- Sex impacts on the metabolome of extremely premature infants (EPI) under real-world clinical antibiotics treatment.
- Antibiotic therapy triggers significant metabolic alterations in male EPI but has a scarce effect in female EPI.
- Sex-related differences are more pronounced in EPI infants treated with penicillins + aminoglycosides, especially in males.
- Female EPI showed more stable metabolic profiles and were less sensitive to pharmacological treatment.
- These findings emphasize the importance of sex awareness in neonatal care.

**Supplementary Information:**

The online version contains supplementary material available at 10.1186/s13293-025-00798-1.

## Background

Sex differences have gained attention in medicine, although they remain frequently overlooked [1–3]. They also occur early at the prenatal stage with Clarke documenting disparities in birth outcomes back to 1786 [4]. Notably, male sex is identified as an independent risk factor for adverse pregnancy outcomes [5]. Since the early 1970s, the term ‘male disadvantage’ has been used to describe the higher incidence of perinatal mortality in male compared to female infants [6]. In contrast, females often exhibit a physiological advantage during the perinatal period, leading to more favourable outcomes in cases of preterm birth [7, 8].

Prematurity, characterized as childbirth occurring before the completion of 37 weeks of gestation, is recorded in 10% of pregnancies each year [9, 10]. Extremely preterm births, defined as those occurring before 28 weeks of gestation, account for approximately 0.66% of all deliveries and these babies are classified as extremely premature infants (EPI) [11]. Mortality and morbidity in preterm babies rise with the early onset of prematurity [12]. Infections and respiratory disorders such as apnea are the most frequent incident clinical conditions in EPI [13–15]. The primary risk factors include prematurity and low birth weight (BW), with male sex representing an additional contributing risk factor [16].

In this context, antibiotics are the most prescribed medications in the first days of life [17], particularly in EPI, of whom up to 90% receive intravenous antibiotics [18, 19]. Such treatments are administered to improve infection control as well as respiratory and neurological outcomes [20, 21]. However, this widespread use exceeds the actual burden of disease, as only 0.5–2% of treated infants have a culture-proven bacterial infection [22]. Such exposure is not without risks: broad-spectrum antibiotics have been associated with early complications, including bronchopulmonary dysplasia, necrotizing enterocolitis, and increased mortality [19, 23]. Although a recent study reported no association between early antibiotic exposure and short-term adverse outcomes in very premature infants (VPI) [24]. Broad-spectrum antibiotics can also alter the gut microbiota, potentially increasing the risk of chronic conditions such as asthma later in childhood and adulthood [25, 26]. Moreover, extensive antibiotic use contributes to the emergence of antibiotic-resistant pathogens [26].

The most commonly used antibiotics are β-lactams and aminoglycosides [27, 28]. These drugs can also affect the mitochondria of eukaryotic cells, potentially leading to adverse effects [19, 29–31]. The interaction of penicillins and aminoglycosides with mitochondria is of particular interest in the field of sex-gender pharmacology, as these cellular powerhouses exhibit numerous sex-related differences at both genetic and functional levels [32–35].

Although antibiotics are widely used in neonates and preterm infants, these populations have been historically excluded from clinical trials, thus leaving pharmacokinetics and pharmacodynamics characterization yet undefined [36, 37]. Currently, pharmacological studies specifically focusing on preterm infants are scarce, and even fewer investigations address EPI [38]. Moreover, only a limited number of studies have explored sex-related differences in drug responses, despite the well-documented sex differences in EPI [39]. Changes in the blood metabolome can be associated with health or disease status and therefore metabolomics can aid to identify molecular signatures early in life, correlate them with biological processes or adverse phenotypes also in response to pharmacological treatments.

Based on these observations, this pilot study aimed to analyse sex differences in the metabolome within a cohort of EPI as a function of antibiotic therapy, using a targeted mass spectrometry (MS)-based approach for the detection of AA and AC in dried blood spots (DBS).

## Methods

### Population

This retrospective study examined a total of 281 EPI identified through the extended newborn screening program in the Campania region (Italy) between 2018 and 2023. The study was approved by the Ethical Committee of the University of Naples Federico II (protocol n. 77/21). Inclusion and exclusion criteria were applied as follows. Infants were excluded if they:

1. born after 28 weeks of gestation;
2. died within 72 h after sample collection owing to severe metabolic imbalance and related complications;
3. were treated with glucose and calcium gluconate solutions;
4. received specific medications (e.g., dopamine, dobutamine, fentanyl, steroids, insulin, caffeine) that could interfere with metabolite analysis or act as surrogate markers of illness;
5. underwent blood transfusions;
6. presented congenital anomalies;
7. received a diagnosis of a metabolic disorder.

After proper selection of the original newborn cohort, we collected data from the MS-based metabolomics analysis of 116 EPI dried blood spots (DBS). All individuals were anonymized. All infants received total parenteral nutrition, consisting of carbohydrates (primarily glucose), proteins (essential and non-essential amino acids), lipids, and a comprehensive mix of vitamins [40].

EPI were stratified for sex and treatment into the following groups:

1. Infants (males and females) who did not receive any pharmacological treatments.
2. Infants (males and females) who received antibiotics, including various classes and combinations of drugs. The principal antibiotics in this group were β-lactams and aminoglycosides, primarily ampicillin and gentamicin.

Within the antibiotic-treated cohort, we further identified a single subgroup composed of infants treated with the penicillins + aminoglycosides combination. This subgroup was then stratified by sex (penicillins + aminoglycosides males and penicillins + aminoglycosides females) to specifically assess sex-related differences in response to this commonly used regimen.

### Sample preparation for metabolomic analysis

Blood samples were collected from the heel of newborns between 48 and 72 h of life as a routine procedure for newborn screening. Blood drops were spotted on a special filter paper and dried overnight at room temperature. DBS were processed and measured by targeted liquid chromatography–tandem mass spectrometry (LC–MS/MS) [40]. Metabolites were extracted from DBS with the addition of 200 µL of methanol containing a mix of stable isotope-labeled standards for AA and AC. Samples were incubated at room temperature for 20 min in agitation and dried under nitrogen flow. Metabolites were derivatized to butyl esters by adding 80 µL of n-butanol/3N HCl at 65 °C for 25 min. Samples were dried and suspended in 300 µL of an acetonitrile/water (70:30) solution acidified with formic acid (0.05%). The analysed compounds were reported in Additional file 1: Table S1.

### Targeted LC–MS/MS analysis

Volumes of 40 µL were introduced by flow injection into the MS system, composed by an API 4000 triple quadrupole (SCIEX, Toronto, Canada) coupled with a 1260 Infinity II HPLC (Agilent Technologies, Waldbronn, Germany). AA and AC detection was performed by precursor ion scan, neutral loss scan, or multiple reaction monitoring (MRM) [41]. Parameters for precursor ion scan analysis of AC were: precursor ion mass: 85.1 Da; polarity: positive; m/z range: 200–560 Da; declustering potential (DP) range: 55–80 V; collision energy (CE) range: 34–60 V. Parameters for neutral loss scan analysis of Ala, Asp, Glu, Met, Phe, Tyr, Val and Xle were: neutral loss mass: 102 Da; polarity: positive; m/z range: 130–280 Da; DP: 45 V; CE: 25 V. Finally, parameters for MRM were: positive polarity; Q1/Q3 (m/z): 132.1/76.0 (Gly), 189.1/70.0 (Orn), 231.2/70.0 (Arg), 232.2/113.1 (Cit) Da; DP: 43 (Gly), 33 (Orn), 60 (Arg), 50 (Cit) volts; CE: 14 (Gly), 33 (Orn), 45 (Arg), 28 (Cit) volts. Concentration values (µM) for each metabolite were obtained with the ChemoView v1.2 software through comparison of the analyte peak area and that of its corresponding internal standard. Accuracy and precision of the MS platform are systematically evaluated for each analytical batch using quality control (QC) samples prepared at four concentration levels (low, mid, high, and very high), supplied by the Centers for Disease Control and Prevention (CDC, Atlanta, GA, USA). Additionally, blanks are incorporated into the sample list sequence to avoid potential carryover and background contamination.

### Statistical and bioinformatic analysis

Descriptive statistics were used to summarize the collected data. Quantitative variables were described as mean and standard deviations (SD) or median and interquartile range (IQR; 25th–75th percentiles), whereas qualitative ones by frequencies and percentages. The Shapiro–Wilk test was performed to assess the normality of distributions. Pearson’s or Spearman’s correlation coefficients were calculated to explore relationships between study variables. Qualitative variables were compared using Pearson’s chi-square test or Fisher’s exact test, whereas quantitative variables were analysed with either the unpaired Student’s t-test or the Mann–Whitney *U* test based on their normal or non-normal distribution. Comparisons between two independent groups (e.g., males vs. females, antibiotic-treated vs. untreated within each sex, sex differences within the penicillins + aminoglycosides subgroup) were performed using the unpaired Student’s t-test or the Mann–Whitney U test, according to data distribution. In line with the study's objective, our analysis focused on bivariate comparisons to examine the specific relationships of interest (comparisons between male antibiotic-treated subjects and female untreated subjects, and vice versa, do not have inherent biological meaning and, therefore, are not considered).

Volcano plots were generated by VolcaNoseR app [42] and used to represent binary comparisons.

Hierarchical cluster analysis based on Gower distance was carried out to identify homogeneous subgroups (i.e., clusters) within the study population. Once distinct clusters were defined, significant differences were evaluated, using one-way analysis of variance (ANOVA).

A *p*-value < 0.05 was considered statistically significant, and all statistical computations were performed using STATA 17 (StataCorp, College Station, TX, USA).

Multivariate analysis of the metabolomic dataset was carried out using MetaboAnalyst 6.0 [43–45]. The data were imported and normalized (log10-transformed and auto-scaled). Partial-least square discriminant analysis (PLS-DA) models were created to investigate the level of variance in the analyzed groups. PLS-DA was carried out with the fivefold cross validation method using five components for the classification, checking the fitness (R2) and the accuracy of the model as performance measures. Hierarchical clustering analysis, heatmaps and Pearson’s correlation were generated with SRplot tool using Euclidean distance [46]. Quantitative Enrichment Analysis (QEA) based on the *globaltest* algorithm of MetaboAnalyst was performed inputting raw metabolite concentrations [47]. QEA used metabolite and lipid pathways from RaMP-DB database (integrating KEGG via HMDB, Reactome, WikiPathways) to highlight significant metabolite sets.

## Results

### Populations

**Fig. 1:**
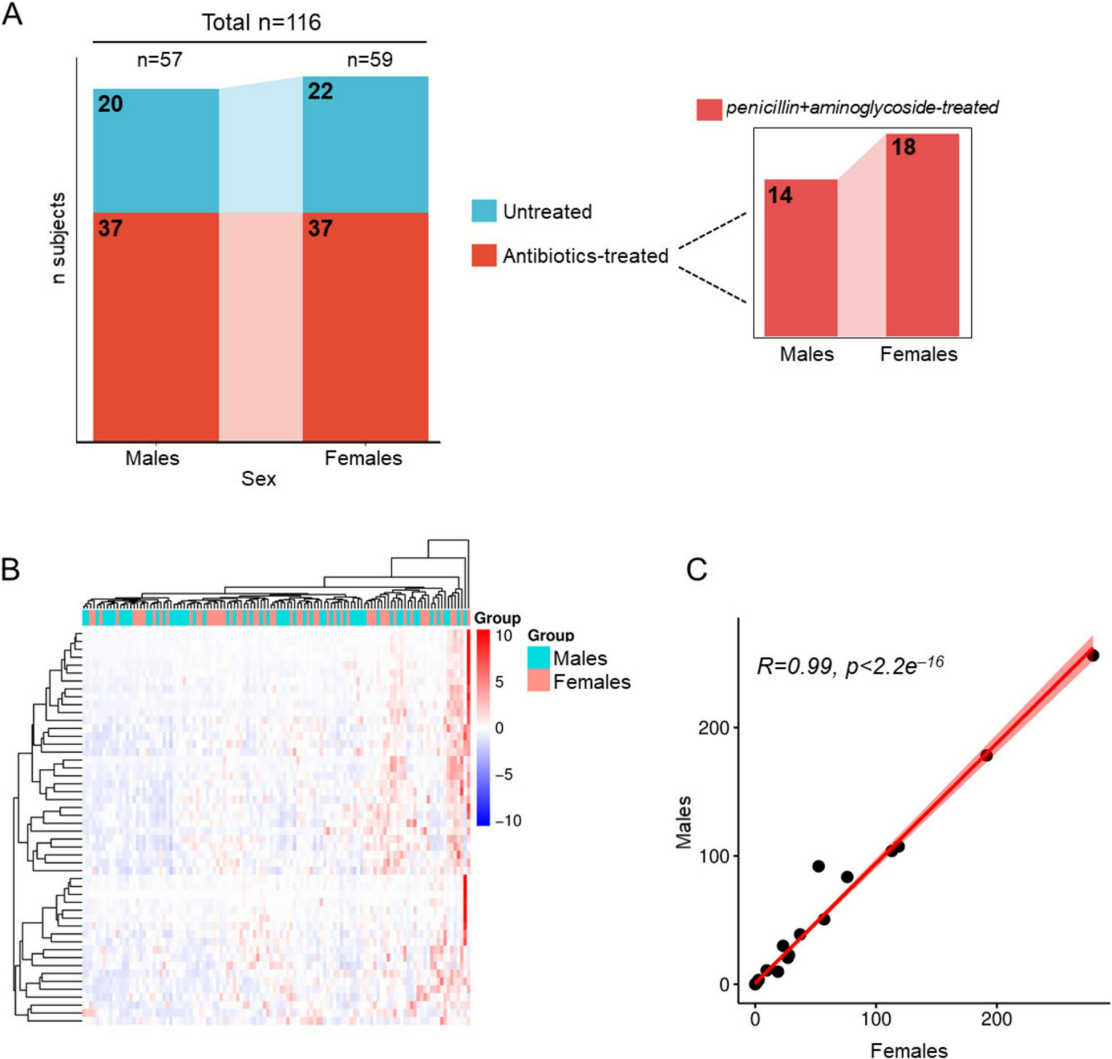
Description and characterization of EPI. **A** Schematic classification of the male and female subgroups stratified according to the antibiotic treatment.** B** Hierarchical clustering applied to the metabolomic dataset to find group classification. **C** Statistical Pearson’s correlation of AA and AC levels performed correlating the mean concentrations for each analyte in both male and female EPI

Due to the low incidence of extremely preterm birth, only 281 EPI were identified over the five-year period; after applying the exclusion criteria, 116 EPI were ultimately included in the study. They were composed by 57 males (49.1%) and 59 females (50.9%), as schematically reported in Fig. 1A. Of these, the 36.2% did not receive any pharmacological treatment (untreated: 20 males; 22 females), the 63.8% received antibiotics (antibiotics-treated: 37 males; 37 females). The antibiotics-treated group was further stratified in EPI treated with the combination of penicillins and aminoglycosides (penicillins + aminoglycosides-treated: 14 males; 18 females). No significant differences in BW and gestational age (GA) were observed between male and female EPI (Table 1).

**Table 1:** BW and GA in the different cohorts of EPI

EPI	BW (g)	GA (weeks)
Untreated males (n = 20)	890.0 (702.5–965.0)	26.0 (26.0–27.0)
Untreated females (n = 22)	737.0 (655.0–820.0)	25.0 (24.0–26.0)
Antibiotics-treated males (n = 37)	830.0 (700.0–930.0)	25.0 (24.7–27.0)
Antibiotics-treated females (n = 37)	810.0 (690.0–950.0)	26.0 (25.0–27.0)

Data are reported as the median (IQR)

### Sex differences in EPI

**Fig. 2:**
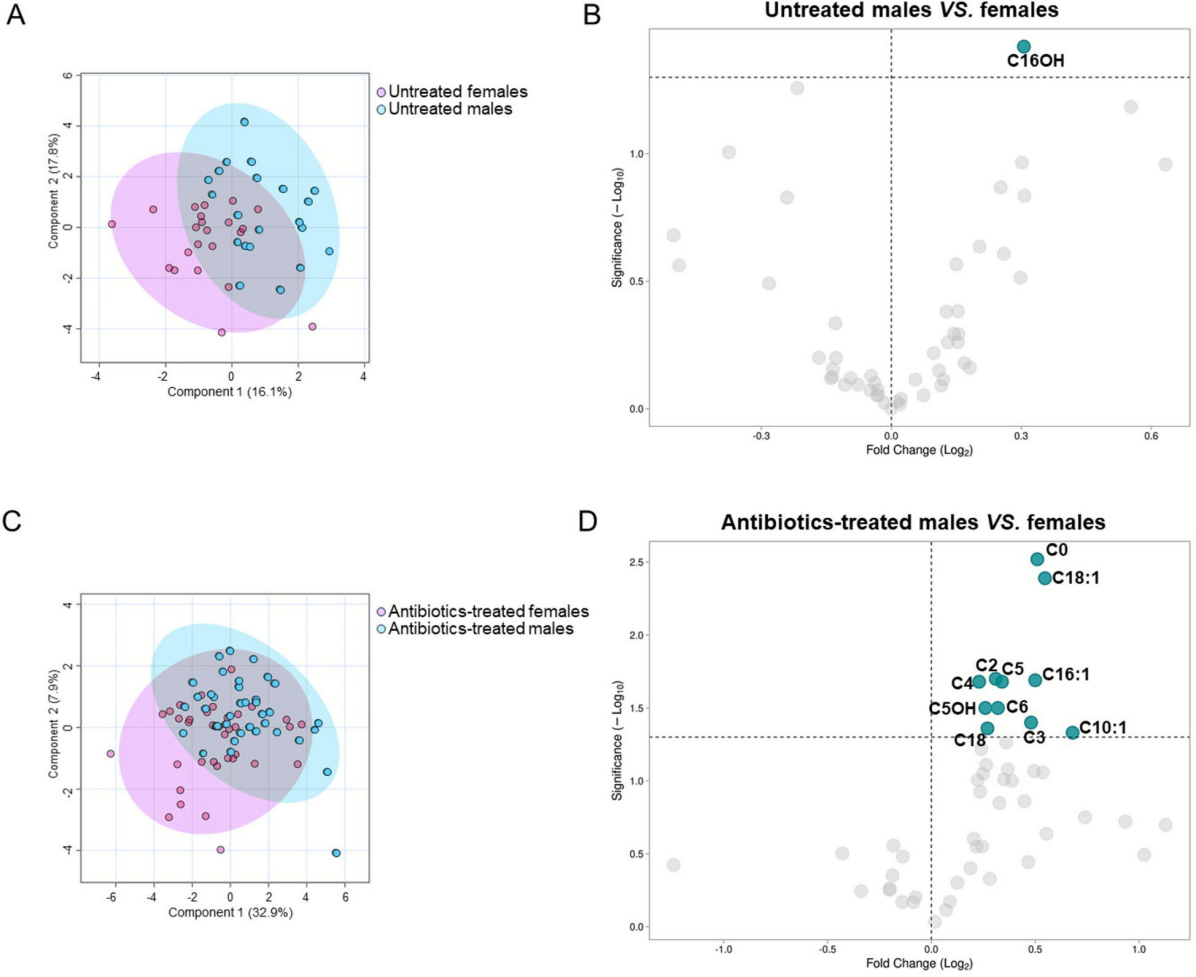
Distribution of metabolomics data and differential analysis of sex differences in the male and female EPI. PLS-DA models (left panels) and corresponding volcano plots (right panels) used to assess the metabolic variance and identify differentially abundant metabolites, respectively, between male and female in the panels **A**, **B**) untreated males *vs* females, **C**, **D**) antibiotics-treated males *vs .* females

When the sex was considered the unique variable, hierarchical clustering failed to discriminate male and female EPI (Fig. 1B). This was further confirmed by Pearson’s correlation analysis, which evidenced a clear overlap of male and female metabolomes (R = 0.99, *p* < 2.2e^–16^) (Fig. 1C). Then, to dissect sex-related metabolic differences in EPI, the metabolome of untreated males and females was compared. PLS-DA showed a small variance between the two groups (Fig. 2A), with C16OH being the only metabolite showing a statistically significant difference (*p* = 0.04) between males and females [median value (IQR): 0.02 (0.02–0.03) µM] than in females [0.02 (0.01–0.03) µM] (Fig. 2B). All the other measured analytes with their abbreviations are reported in Additional file 1: Table S1.

Male and female EPI who received empirical antibiotic therapy displayed greater variance as shown by the PLS-DA model (Fig. 2C) revealing significant differences in the levels of 11 AC (Fig. 2D and Table 2), while AA levels did not diverge between sexes. Specifically, C0, C2, C3, C4, C5, C6, C5OH, C10:1, C16:1, C18, and C18:1were significantly higher in males (Fig. 2D and Table 2).

**Table 2:** Statistically significant sex differences in blood metabolite concentrations (µM) between male and female antibiotics-treated EPI

Variables	Antibiotics-treated males (n = 37)	Antibiotics-treated females (n = 37)	*p*-value
C0	35.49 (27.83–57.60)	24.89 (17.70–36.46)	0.003
C2	25.91 (20.72–37.07)	20.88 (15.44–29.38)	0.02
C3	3.06 (2.12–3.92)	2.19 (1.51–3.17)	0.035
C4	0.54 (0.44–0.89)	0.46 (0.34–0.53)	0.02
C5	0.38 (0.29–0.70)	0.30 (0.24–0.38)	0.02
C6	0.05 (0.04–0.08)	0.04 (0.03–0.05)	0.03
C5OH	0.18 (0.15–0.21)	0.15 (0.11–0.18)	0.03
C10:1	0.08 (0.06–0.13)	0.05 (0.04–0.15)	0.047
C16:1	0.17 (0.12–0.21)	0.12 (0.08–0.19)	0.02
C18:1	1.52 (1.13–1.84)	1.04 (0.75–1.47)	0.004
C18	0.71 (0.59–0.92)	0.59 (0.50–0.77)	0.044

Variables are summarized as median (25th–75th percentiles)

**Fig. 3:**
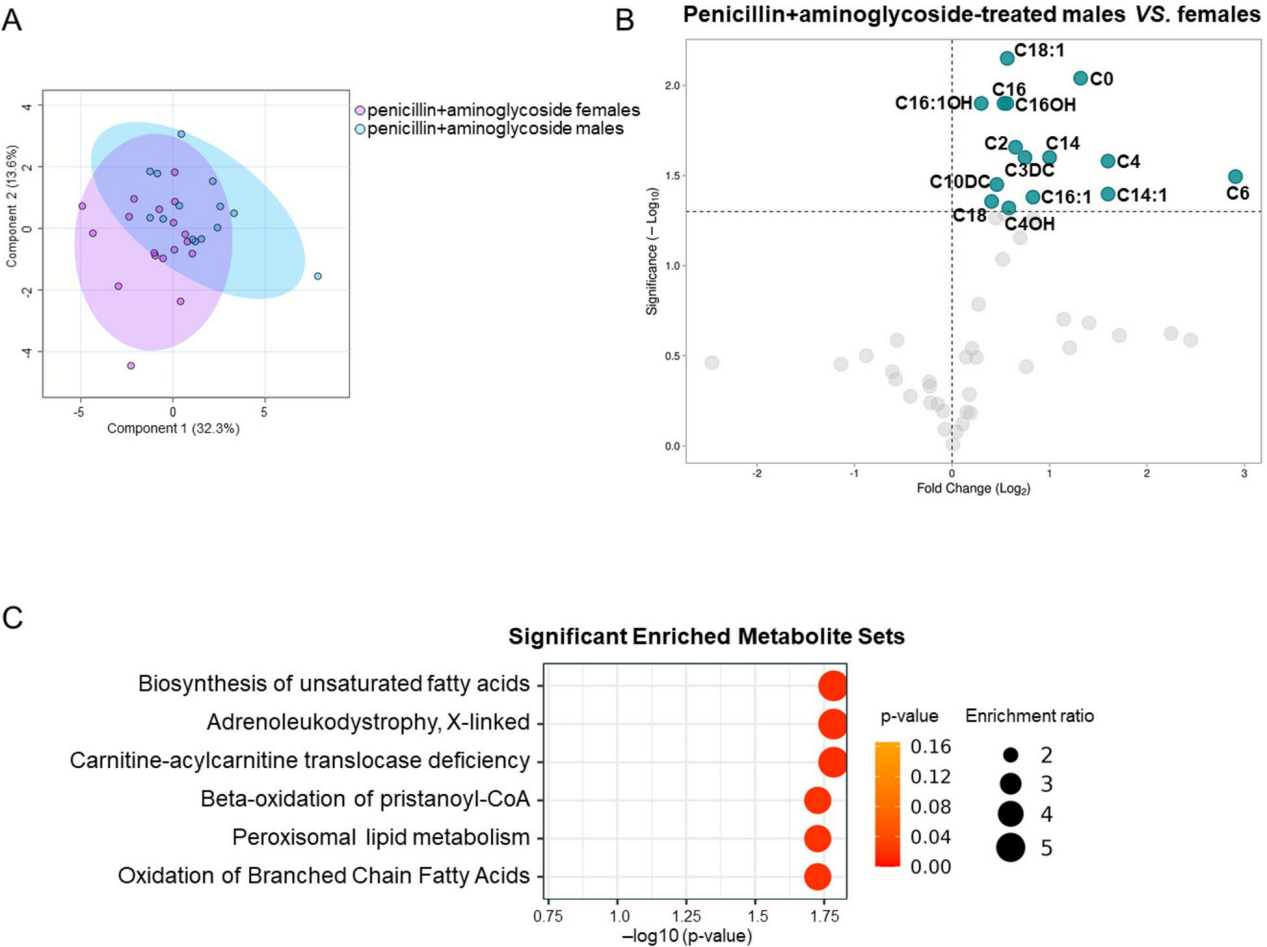
Distribution of metabolomics data and differential analysis of sex-related differences in combined penicillins + aminoglycosides treatment subgroups. **A** PLS-DA model was generated to assess the metabolic variance of groups. **B** Volcano plot shows the differentially abundant metabolites in penicillins + aminoglycoside-treated males vs females. **C** Quantitative enrichment analysis was performed to highlight significant metabolite sets based on metabolite concentrations of male and female subgroups

Because most of EPI were treated with a combination of penicillins + aminoglycosides we further focused on this subgroup of subjects. PLS-DA analysis revealed high group variance between sexes (Fig. 3A). Differential analysis confirmed that some sex differences were common to all class-antibiotics group (Fig. 2D), while other sex differences emerged. In particular, C0, C2, C4, C6, C16:1, C18, and C18:1 remained significantly higher in males who received the penicillins + aminoglycosides combination, while C3, C5, C5OH, and C10:1 did not diverge anymore between male and female EPI. Moreover, eight AC showed significant differences that were not observed in the antibiotic-treated group. Specifically, C3DC, C14:1, C14, C16, C10DC, C16OH, C4OH, and C16:1OH were significantly higher in penicillins + aminoglycosides-treated males than females (Fig. 3B). All the other parameters did not show statistical difference. The complete set of statistical analysis performed in the pencillins + aminoglycosides groups is showed in Additional file 1: Table S2.

Moreover, quantitative enrichment analysis (QEA) was performed to highlight significant metabolite sets based on metabolite concentrations. QEA showed consistent changes among compounds within the same biological pathway, enriching terms connected with lipid metabolism, including the biosynthesis and the oxidation of fatty acids and carnitine/AC transport (Fig. 3C).

### Intra-sex metabolic variability in EPI

**Fig. 4:**
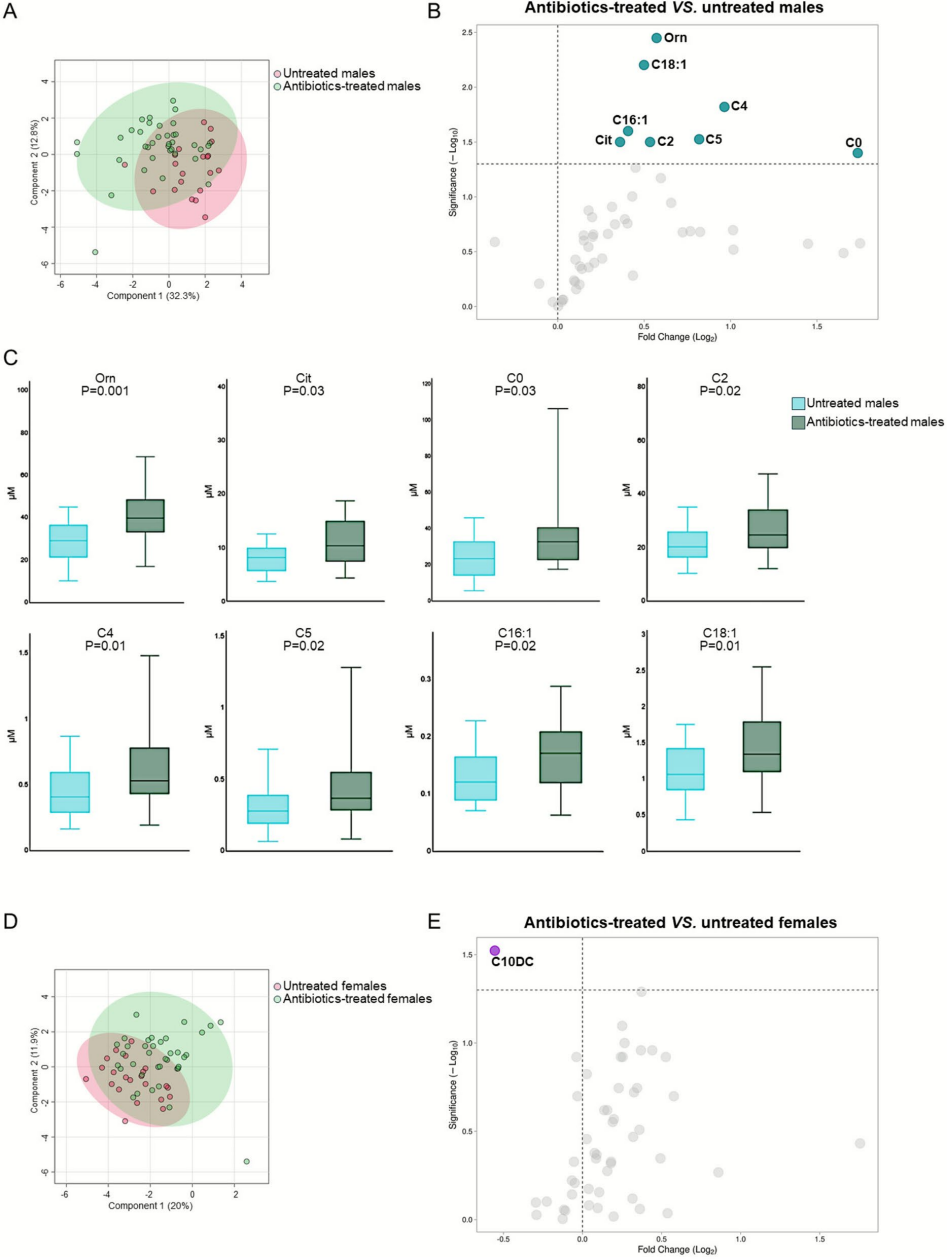
Distribution of metabolomics data and differential analysis of intra-sex differences in antibiotics-treated and untreated male and female EPI. **A** PLS-DA model and **B** volcano plot to assess the metabolic variance and identify differentially abundant metabolites, respectively, in antibiotics-treated *vs.* untreated males. **C** Values of concentrations and statistical significance for individual metabolites significant from volcano plot analysis. **D** PLS-DA model and **E** volcano plot to assess the metabolic variance and identify differentially abundant metabolites, respectively, in antibiotics-treated *vs.* untreated females

In males, antibiotics treatments impacted on metabolites concentrations showing pronounced variance with respect to the untreated group (Fig. 4A). Specifically, antibiotics administration significantly increased the levels of two AA (Orn and Cit) and six AC (C0, C2, C4, C5, C16:1, C18:1; Fig. 4B and C). The set of statistical analysis was reported in Additional file 1: Table S3. By contrast, in female EPI, empiric antibiotics treatment had a limited effect on metabolomic variations (Fig. 4D), with C10DC being the only molecule that differed significantly in antibiotics-treated [median value (IQR): 0.20 (0.10–0.33) µM] *versus* untreated females [0.33 (0.16–0.56) µM] (*p* = 0.03) (Fig. 4E). All other measured metabolites are reported in Additional file 1: Table S3.

### Correlation and cluster analysis of metabolic variables in EPI

**Fig. 5:**
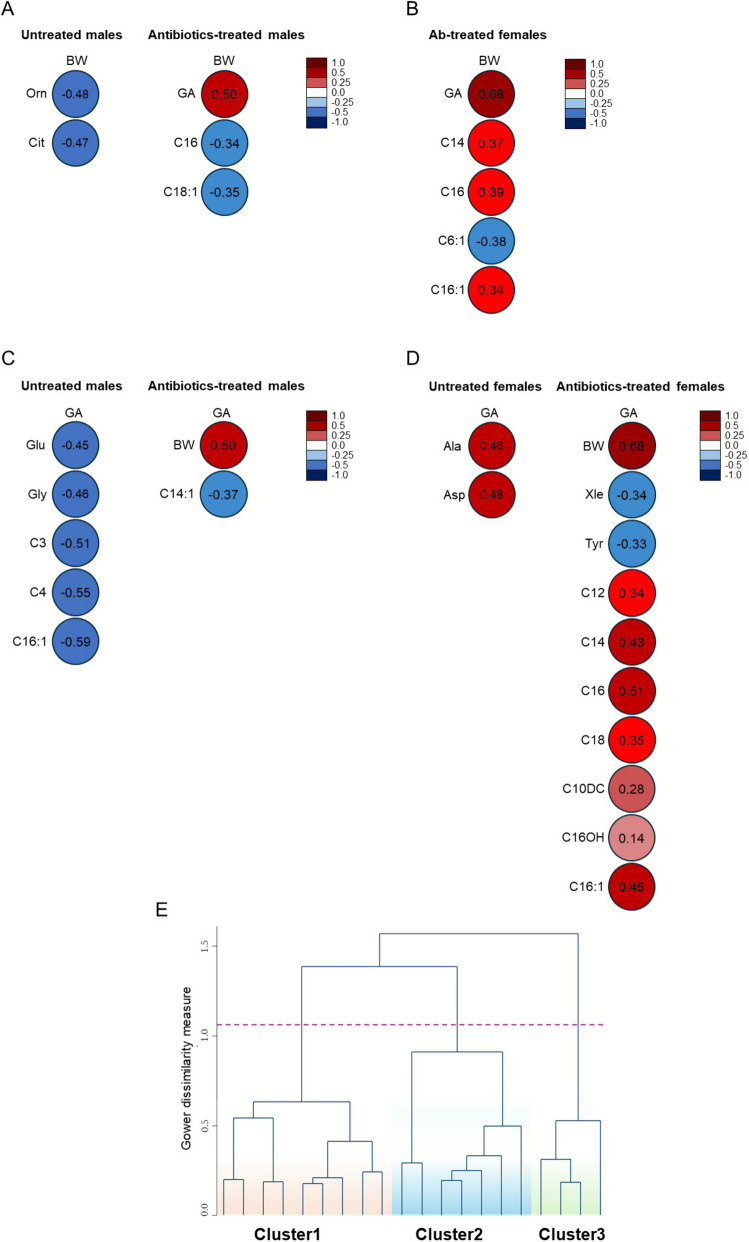
Correlation heatmaps of statistically significant associations between **A**, **B** birth weight (BW) or **C**, **D** gestational age (GA) and the analysed metabolites in male and female EPI, respectively, under different treatment conditions. Circles represent Pearson’s correlation coefficients: blue circles indicate negative correlations and red circles denote positive correlations in a scale ranging from –1.0 to 1.0. **E** Dendrogram for hierarchical cluster analysis of metabolomic data

Correlation analysis was performed to assess the association between BW or GA with AA and AC. In untreated male EPI, Orn and Cit were negatively related with BW (Fig. 5A), whereas GA was inversely correlated with Glu, Gly, C3, C4, and C16:1 (Fig. 5C).

In antibiotics-treated male EPI, BW was positively correlated with GA and negatively correlated with C16 and C18:1 (Fig. 5A), whereas GA was negatively correlated with C14:1 (Fig. 5C).

Untreated female EPI showed no significant correlations with BW, while a significant positive correlation was observed for GA with Ala and Asp (Fig. 5B, D). In antibiotics-treated female EPI, BW was positively associated with GA, C14, C16, and C16:1, and negatively with C6:1 (Fig. 5B). GA also correlated positively with BW and several AC including C12, C14, C16, C18, C10DC, C16OH, and C16:1, while showing negative correlations with Xle and Tyr (Fig. 5D).

Full data regarding correlation analysis, including correlation coefficients and corresponding *p*-values, are reported in the Additional file 1: Table S4–S5.

Results from cluster analysis showed three distinct clusters (Fig. 5E and Table 3). The clustering pattern was independent of sex allocation, indicating a uniform distribution, while subgroups showed significant differences when stratified by treatment. Cluster 1 was predominantly represented by untreated EPI, whereas Clusters 2 and 3 included antibiotic-treated EPI with an equal distribution of sexes.

**Table 3:** Comparison among clusters

Variables		Cluster 1 (n: 45)	Cluster 2 (n: 62)	Cluster 3 (n: 9)	*p-value*
Male, n (%)		23 (51.1)	29 (46.8)	5 (55.6)	0.81
Treatment, n (%)	Untreated neonates	42 (93.3)	0 (0.0)	0 (0.0)	< 0.0001
	Antibiotics-treated neonates	3 (6.7)	62 (100.0)	9 (100.0)	
Group, n (%)	Untreated males	20 (44.4)	0 (0.0)	0 (0.0)	< 0.0001
	Untreated females	22 (48.9)	0 (0.0)	0 (0.0)	
	Antibiotics males	3 (6.7)	29 (46.8)	5 (55.6)	
	Antibiotics females	0 (0.0)	33 (53.2)	4 (44.4)	
BW		822.5 (188.1)	826.8 (179.9)	746.7 (179.9)	0.46
GA		26 (25–27)	26 (25–27)	25 (24–27)	0.60
Ala		129.1 (103.8–164.3)	129.3 (97.5–166.0)	105.9 (87.5–137.7)	0.33
Val		92.2 (74.6–114.2)	94.0 (81.1–132.0)	74.2 (51.6–75.5) ^*,^^°^	0.02
Xle		102.1 (70.5–124.5)	101.7 (79.7–120.7)	99.0 (89.0–100.8)	0.78
Met		17.8 (11.5–27.5)	17.2 (11.8–22.4)	15.8 (11.1–20.7)	0.95
Phe		44.7 (39.6–63.7)	46.4 (41.1–55.6)	47.5 (42.7–49.5)	0.94
Tyr		46.3 (31.4–136.9)	59.4 (37.7–78.8)	67.5 (30.6–169.0)	0.88
Asp		21.5 (16.2–26.5)	20.5 (16.8–28.7)	14.2 (10.1–17.1)	0.07
Glu		170.1 (129.8–203.4)	173.4 (148.2–212.4)	177.9 (141.6–209.7)	0.41
Gly		268.3 (220.5–304.1)	228.6 (203.4–308.5)	257.0 (230.0–301.4)	0.33
Orn		31.5 (21.3–40.4)	38.4 (32.5–44.3)	37.2 (26.2–44.6)	0.08
Cit		8.7 (6.3–11.8)	9.4 (7.0–11.9)	10.7 (7.7–12.2)	0.67
Arg		7.9 (3.9–11.2)	7.8 (5.5–10.7)	6.1 (4.2–7.0)	0.52
C0		23.2 (17.6–37.0)	28.6 (19.9–38.2)	462.2 (356.9–543.7) ^**,^^°°^	0.0001
C2		19.6 (16.0–24.1)	22.2 (16.7–27.7)	68.0 (53.0–80.5) ^**,^^°°^	0.0001
C3		2.0 (1.5–3.5)	2.3 (1.7–3.2)	6.9 (5.5–8.8) ^**,^^°°^	0.0001
C4		0.41 (0.32–0.56)	0.47 (0.35–0.57)	2.27 (1.55–2.3) ^**,^^°°^	0.0001
C5:1		0.03 (0.02–0.04)	0.03 (0.02–0.03)	0.05 (0.04–0.05) ^*,^^°^	0.001
C5		0.32 (0.22–0.42)	0.31 (0.25–0.38)	1.72 (1.28–1.85) ^**,^^°°^	0.0001
C6		0.04 (0.03–0.05)	0.04 (0.03–0.05)	0.31 (0.26–0.52) ^**,^^°°^	0.0001
C5OH		0.15 (0.12–0.19)	0.16 (0.11–0.18)	0.23 (0.2–0.24) ^*,^^°^	0.004
C8		0.08 (0.06–0.13)	0.09 (0.07–0.12)	0.44 (0.38–0.57) ^**,^^°°^	0.0001
C3DC		0.04 (0.03–0.05)	0.04 (0.03–0.05)	0.1 (0.09–0.13) ^**,^^°°^	0.0001
C10:1		0.06 (0.04–0.1)	0.06 (0.05–0.09)	0.37 (0.29–0.49) ^**,^^°°^	0.0001
C10		0.06 (0.04–0.09)	0.06 (0.04–0.08)	0.36 (0.33–0.66) ^**,^^°°^	0.0001
C4DC		0.10 (0.08–0.16)	0.11 (0.09–0.13)	0.17 (0.12–0.19) ^*^	0.06
C5DC		0.05 (0.04–0.06)	0.05 (0.04–0.07)	0.22 (0.18–0.32) ^**,^^°°^	0.0001
C12:1		0.03 (0.02–0.05)	0.03 (0.03–0.04)	0.23 (0.20–0.25) ^**,^^°°^	0.0001
C12		0.08 (0.06–0.13)	0.08 (0.06–0.10)	0.21 (0.20–0.28) ^*,^^°°^	0.0001
C6DC		0.04 (0.03–0.07)	0.04 (0.03–0.06)	0.07 (0.05–0.08)	0.17
C14:2		0.07 (0.05–0.09)	0.07 (0.05–0.10)	0.15 (0.12–0.19) ^*,^^°^	0.0002
C14:1		0.11 (0.08–0.15)	0.12 (0.09–0.17)	0.51 (0.43–0.54) ^**,^^°°^	0.0001
C14		0.13 (0.1–0.2)	0.14 (0.11–0.19)	0.26 (0.23–0.27) ^*,^^°^	0.001
C8DC		0.03 (0.02–0.04)	0.03 (0.02–0.04)	0.06 (0.04–0.06) ^*,^^°^	0.004
C16:1		0.11 (0.09–0.16)	0.13 (0.1–0.19)	0.26 (0.22–0.27) ^**,^^°^	0.0001
C16		0.95 (0.77–1.48)	1.09 (0.75–1.46)	1.53 (1.38–1.89) ^*,^^°^	0.044
C10DC		0.27 (0.16–0.45)	0.22 (0.13–0.35)	0.25 (0.23–0.35)	0.25
C16OH		0.02 (0.01–0.03)	0.02 (0.02–0.03)	0.06 (0.05–0.07) ^**,^^°°^	0.0001
C18:1		0.96 (0.62–1.33)	1.19 (0.86–1.63)	2.52 (1.98–2.87) ^**,^^°°^	0.0001
C18		0.61 (0.45–0.84)	0.64 (0.51–0.82)	0.94 (0.68–1.00) ^*,^^°^	0.03
C18:1OH		0.03 (0.02–0.03)	0.03 (0.02–0.04)	0.06 (0.06–0.07) ^**,^^°^	0.0001
C4OH		0.11 (0.09–0.15)	0.13 (0.1–0.17)	0.25 (0.23–0.35) ^**,^^°^	0.0001
C6OH		0.04 (0.03–0.04)	0.04 (0.03–0.05)	0.05 (0.05–0.07) ^*,^^°^	0.001
C6:1		0.03 (0.02–0.05)	0.02 (0.02–0.03)	0.05 (0.04–0.06) ^*,^^°^	0.006
C8:1		0.07 (0.04–0.14)	0.07 (0.04–0.10)	0.15 (0.12–0.19) ^*,^^°^	0.005
C10:2		0.04 (0.02–0.05)	0.03 (0.02–0.06)	0.08 (0.06–0.09) ^*,^^°^	0.001
C12OH		0.02 (0.01–0.03)	0.02 (0.02–0.03)	0.06 (0.06–0.08) ^**,^^°^	0.0001
C14OH		0.02 (0.02–0.03)	0.03 (0.02–0.03)	0.05 (0.05–0.07) ^*,^^°^	0.0004
C16:1OH		0.04 (0.03–0.04)	0.04 (0.03–0.05)	0.08 (0.07–0.08) ^**,^^°°^	0.0001
C18OH		0.02 (0.01–0.02)	0.02 (0.01–0.02)	0.03 (0.03–0.03) ^*,^^°^	0.0002
C18:2		0.33 (0.20–0.43)	0.32 (0.23–0.48)	0.34 (0.30–0.46)	0.83

Quantitative variables are summarized as mean (SD) or median (25th-75th percentiles). Post-hoc comparison analysis: * Cluster 1 *vs.* Cluster 3; °Cluster 2 *vs.* Cluster 3, indicate a *p* < 0.05. **Cluster 1 *vs.* Cluster 3; °°Cluster 2 *vs.* Cluster 3, indicate a *p* < 0.001. AA and AC concentrations are reported as µM

Despite being smaller in size, Cluster 3 was characterized by significantly elevated concentrations of most of the analyzed AC compared to Clusters 1 and 2. Specifically, all AC apart from C6DC, C10DC, and C18:2 were significantly increased in Cluster 3 versus Clusters 1 and 2. No significant differences were detected in AA, except for Val, which was lower in Cluster 3 compared to Clusters 1 and 2.

## Discussion

While the importance of sex and gender in health and medicine has been increasingly recognized in adult populations [1–3], recent evidence suggests that sex differences are also relevant during prenatal and neonatal life [48–50]. In this context, metabolomics has emerged as a powerful tool to characterize dynamic biochemical phenotypes shaped by gene-environment interactions, allowing the identification of disease-specific metabolic profiles and pharmacological response [51]. Despite this potential, the application of metabolomics in newborns, particularly in preterm populations, remains limited, even as interest in the field grows [52, 53]. Importantly, biological sex continues to be frequently overlooked in both metabolomics studies [51] and neonatal research [54], highlighting a critical gap in current research.

Indeed, although some studies have assessed metabolite changes in VPI [55–59], only very few research investigated the effects of sex on blood metabolome. As example, VPI and moderate-to-late preterm infants showed variations in blood metabolomic profiles that are dependent on BW, GA, and nutritional factors [60]. Moreover, it has been demonstrated that the metabolome of human amniocytes is sex-dependent, reflecting patterns observed in neonatal populations [50]. 

In preterm neonates (24–32 weeks), recent evidence suggests a sex-dependent response to sepsis [61]. According to literature [17, 18, 22, 26, 61], treating Gram-negative bacterial sepsis remains challenging and often requires the empirical use of broad-spectrum antibiotics. While necessary, this approach carries some risks, including disruption of the gut microbiome and the emergence of antibiotic resistance [26]. Importantly, the alteration of gut microbiome in infants elevates the risks of chronic diseases in adulthood [26]. In other words, the empirical antibiotic treatment is essential in preterm infants care due to the high burden of infections and neonatal sepsis [19, 27, 62]. However, minimizing unnecessary exposure is crucial due to the substantial risk of adverse effects [63], also including phenomena of increased antibiotic resistance due to early antibiotic use [64].

Building on these evidences, this pilot study incorporated sex as a biological variable in the metabolomic profiling of a highly critical neonatal population in a real-life clinical context, comprising EPI treated and untreated with antibiotics, allowing us to evaluate baseline metabolic differences as well as antibiotics-induced variations. Untreated EPI show very similar metabolic profiles in males and females, with the only significant increase of C16OH in males, indicating that sex begins to influence mitochondrial function already at low GA [65]. Previous findings revealed sex-related differences predominantly in AA levels among VPI, and in AC levels in moderate-to-late preterm infants [60]. These observations suggest that sex differences in blood AA and AC homeostasis are dependent on GA. This underscores the critical role of GA in shaping the emergence and development of sex-dependent metabolic differences.

However, exposure to antibiotics dramatically increases sex-related differences in male EPI, and this effect is further amplified by the combination of penicillins + aminoglycosides, leading to an increase in several AC in males, potentially reflecting inhibition of fatty acid oxidation and altered lipid metabolism. Accordingly, commonly used antibiotics in neonates are known to cause mitochondrial dysfunction in eukaryotic cells [19, 29–31, 66]. For example, pivampicillin may induce carnitine deficiency [66]; penicillin and its derivatives promote oxidative stress [29]; β-lactams can impair fatty acid β-oxidation by inhibiting the mitochondrial carnitine/AC transporter [30]; gentamicin contributes to mitochondrial toxicity by disrupting respiratory chain function and collapsing mitochondrial membrane potential [31], as well as lysosomal membranes [67]. These mechanisms help to explain why antibiotic treatment more significantly affects AC than AA.

In addition, these effects on male EPI are also observed when antibiotics-treated males were compared to untreated ones, suggesting an enhanced effect on the male but not female EPI metabolome.

These data suggest that male prematures’ metabolome is significantly more susceptible to variations induced by antibiotics-based therapies. Currently, at best of our knowledge, it is not known whether adverse drug reactions in EPI are sex-dependent. However, the antibiotic targets on eukaryotic cells could be linked to such reactions, and the sex differences induced by antibiotics treatment make this possibility biologically plausible because male and female mitochondria present numerous sex differences [34, 35, 68–70].

Correlation analysis revealed only a few associations in untreated EPI; however, antibiotics therapy increased the number of correlations, with a greater effect in male compared to female EPI. Moreover, hierarchical clustering based on metabolomic profiles identified three distinct clusters independent of sex distribution but associated with treatment, with untreated EPI primarily clustering in cluster 1 and antibiotic-treated neonates distributed across clusters 2 and 3. Interestingly, cluster 3, although smaller in size, was characterized by a global increase in AC (except C6DC, C10DC, and C18:2) and a selective reduction in Val, suggesting a distinct metabolic phenotype potentially linked to antibiotic exposure and altered energy metabolism.

Untreated infants exhibit a significant increase in pathways involved in short-chain fatty acid biosynthesis compared to antibiotic-treated ones [71]. However, at the moment it is not possible to exclude that mitochondrial alterations are linked to antibiotics induced changes in gut microbiome [72]. Interestingly, sex-related variations have been identified in microbiome [71, 73].

In summary, the blood metabolome of untreated male and female EPI is very similar; however, it is susceptible to antibiotics treatments, with the female metabolome being less affected than the male metabolome. These findings indicate that sex differences are strongly amplified by antibiotic treatment, whereas baseline differences are minimal, implying that, within the limits of our dataset, the male disadvantage in EPI may be pharmacologically enhanced.

In conclusion, this pilot study further confirms that metabolomic analyses are essential for understanding the influence of sex on pharmacological responses, even in EPI, a relatively understudied population. Moreover, our findings suggest that antibiotics exert pharmacodynamic effects at the mitochondrial level, which are strongly modulated by sex. These insights are crucial for advancing neonatal care and developing personalized therapeutic strategies for EPI, and provide a foundation for larger clinical trials.

### Strengths and limitations

Pharmacological responses to antibiotics in EPI are not fully understood, and it remains unclear whether they are influenced by sex. To date, only a few metabolomic studies have been conducted in this population, and biological sex has been largely overlooked in metabolomics-based investigations [51]. Despite the well-established evidence that male sex is an independent risk factor for major morbidities and mortality, particularly in preterm infants [74]. This pilot study extends previous findings, mainly derived from less preterm cohorts, by confirming the existence of sex-dependent metabolic phenotypes and demonstrating their relevance in EPI [55, 60, 75].

The main innovation of this retrospective pilot study is its explicit focus on sex-dependent differences in the metabolome of EPI, and on how antibiotic-induced metabolic alterations vary by sex in this highly vulnerable population. While antibiotics remain indispensable in the management of preterm newborns, minimizing unnecessary exposure is crucial given their well-documented risks of adverse effects [63, 64].

A key limitation of this study is that the analysis included only a restricted category of antibiotics, without the possibility of exploring mechanisms related to other agents. However, this reflects real-world practice, as β-lactams and aminoglycosides are the most widely prescribed antibiotics in the Campania region, as they are worldwide [27, 28]. This represents both a strength and a limitation: while our findings are grounded in actual clinical use, they may have been influenced by differences in physicians’ decisions, particularly in the absence of culture-proven infections. Moreover, our study design does not allow us to determine whether the sex-related metabolic differences observed during antibiotic treatment persist after treatment discontinuation, as no longitudinal or post-treatment follow-up samples were available. Future prospective studies with serial sampling will be required to clarify whether these differences are transient or may have longer-term consequences.

Another limitation lies in the use of a targeted LC–MS/MS metabolomics approach, which by design restricts the analysis to a predefined set of metabolites and therefore limits the investigation of broader metabolic pathways. Untargeted metabolomic strategies could instead provide a more comprehensive overview of sex-related metabolic differences in preterm infants by capturing a wider range of molecular features.

Finally, our results underscore that metabolomic studies are essential for evaluating pharmacological responses while taking into account the influence of sex on biological processes.

Therefore, future studies with larger and statistically powered designs are warranted to confirm and extend these results.

## Supplementary Information

Additional file 1

## Data Availability

All data generated or analysed during this study are included in this published article and its additional files.

## Abbreviations

AA: Amino acids
AC: Acylcarnitines
ANOVA: Analysis of variance
BW: Birth weight
DBS: Dried blood spot
EPI: Extremely premature infants
GA: Gestational age
HPLC: High-performance liquid chromatography
IQR: Interquartile range
LC–MS/MS: Liquid chromatography–tandem mass spectrometry
MRM: Multiple reaction monitoring
PLS-DA: Partial-least square discriminant analysis
QC: Quality controls
QEA: Quantitative enrichment analysis
SD: Standard deviation
VPI: Very premature infants
