# Supplementary material for: Sex differences in the blood metabolome of extremely preterm infants: a pilot study on the impact of antibiotic therapy
Source: Biol Sex Differ. 2025 Dec 6;17:5. doi: 10.1186/s13293-025-00798-1 (PMC12798127; doi:10.1186/s13293-025-00798-1)
Supplement: Supplementary file 1 — Additional file 1 [file 13293_2025_798_MOESM1_ESM.docx]

**Supplementary Table S1. All the measured variables in untreated male and female EPI**

| **Variables** | **Untreated male EPI**  **(n=20)** | **Untreated female EPI**  **(n=22)** | **p-value** |
| --- | --- | --- | --- |
| BW (Birth weight) | 890 (702.5-965.0) | 737 (655-820) | 0.15 |
| GA (Gestational age) | 26 (26-27) | 25 (24-26) | 0.06 |
| Ala (Alanine) | 118.72 (96.86-171.14) | 139.04 (110.01-158.72) | 0.45 |
| Val (Valine) | 84.24 (69.40-129.31) | 93.14 (79.41-106.61) | 0.99 |
| Xle (Isoleucine+Leucine) | 93.65 (67.62-116.63) | 103.72 (72.08-124.12) | 0.72 |
| Met (Methionine) | 14.50 (10.25-21.52) | 17.10 (11.31-27.49) | 0.49 |
| Phe (Phenylalanine) | 44.53 (38.02-51.82) | 44.49 (39.60-72.68) | 0.53 |
| Tyr (Tyrosine) | 42.29 (27.94-78.85) | 45.21 (32.40-136.91) | 0.32 |
| Asp (Aspartate) | 21.62 (15.80-24.33) | 20.75 (14.94-27.68) | 0.97 |
| Glu (Glutamate) | 166.27 (125.85-204.39) | 156.53 (127.93-197.84) | 0.85 |
| Gly (Glycine) | 227.98 (196.22-287.29) | 288.42 (229.03-304.13) | 0.05 |
| Orn (Ornithine) | 28.90 (21.16-36.26) | 32.96 (21.32-54.07) | 0.26 |
| Cit (Citrulline) | 8.11 (5.64-9.87) | 8.51 (6.47-11.85) | 0.31 |
| Arg (Arginine) | 7.81 (4.69-9.82) | 7.11 (2.91-10.71) | 0.58 |
| C0 (Carnitine) | 26.24 (17.80-38.74) | 19.66 (17.10-31.49) | 0.48 |
| C2 (Acetylcarnitine) | 20.79 (16.49-28.43) | 18.84 (14.67-20.45) | 0.23 |
| C3 (Propionylcarnitine) | 2.68 (1.79-3.97) | 1.95 (1.32-2.63) | 0.14 |
| C4 (Butyrylcarnitine) | 0.40 (0.29-0.63) | 0.41 (0.32-0.47) | 0.89 |
| C5:1 (Tiglylcarnitine) | 0.02 (0.02-0.04) | 0.03 (0.02-0.03) | 0.83 |
| C5 (Valerylcarnitine) | 0.28 (0.20-0.41) | 0.32 (0.22-0.39) | 0.68 |
| C6 (Hexanoylcarnitine) | 0.05 (0.03-0.07) | 0.04 (0.04-0.04) | 0.28 |
| C5OH (Hydroxyvalerylcarnitine) | 0.15 (0.12-0.19) | 0.15 (0.11-0.18) | 0.43 |
| C8 (Octanoylcarnitine) | 0.09 (0.06-0.16) | 0.07 (0.06-0.10) | 0.21 |
| C3DC (Malonylcarnitine) | 0.04 (0.03-0.05) | 0.03 (0.03-0.05) | 0.21 |
| C10:1 (Decenoylcarnitine) | 0.06 (0.05-0.09) | 0.05 (0.04-0.09) | 0.33 |
| C10 (Decanoylcarnitine) | 0.06 (0.04-0.10) | 0.05 (0.04-0.08) | 0.51 |
| C4DC (Methylmalonylcarnitine) | 0.11 (0.08-0.17) | 0.09 (0.08-0.16) | 0.51 |
| C5DC (Glutarylcarnitine) | 0.06 (0.04-0.07) | 0.05 (0.04-0.06) | 0.48 |
| C12:1 (Dodecenoylcarnitine) | 0.03 (0.02-0.05) | 0.03 (0.02-0.05) | 0.76 |
| C12 (Dodecanoylcarnitine) | 0.08 (0.06-0.14) | 0.07 (0.05-0.17) | 0.85 |
| C6DC (Methylglutarylcarnitine) | 0.04 (0.03-0.07) | 0.04 (0.03-0.06) | 0.82 |
| C14:2 (Tetradecadienoylcarnitine) | 0.07 (0.06-0.09) | 0.06 (0.04-0.08) | 0.18 |
| C14:1 (Tetradecenoylcarnitine) | 0.13 (0.09-0.17) | 0.10 (0.07-0.14) | 0.26 |
| C14 (Tetradecanoylcarnitine) | 0.16 (0.12-0.23) | 0.13 (0.10-0.18) | 0.19 |
| C8DC (Suberylcarnitine) | 0.04 (0.02-0.04) | 0.03 (0.02-0.04) | 0.58 |
| C16:1 (Hexadecenoylcarnitine) | 0.12 (0.09-0.16) | 0.10 (0.09-0.16) | 0.69 |
| C16 (Hexadecanoylcarnitine) | 1.07 (0.79-1.55) | 1.00 (0.64-1.48) | 0.54 |
| C10DC (Decanedioylcarnitine) | 0.25 (0.18-0.38) | 0.33 (0.16-0.56) | 0.41 |
| C16OH (Hydroxyhexadecanoylcarnitine) | 0.02 (0.02-0.03) | 0.02 (0.01-0.03) | 0.04 |
| C18:1 (Octadecenoylcarnitine) | 1.06 (0.85-1.42) | 0.94 (0.56-1.30) | 0.54 |
| C18 (Octadecanoylcarnitine) | 0.66 (0.46-0.88) | 0.62 (0.44-0.81) | 0.64 |
| C18:1OH (Hydroxyoctadecenoylcarnitine) | 0.03 (0.02-0.03) | 0.02 (0.02-0.03) | 0.31 |
| C4OH (Hydroxybutyrylcarnitine) | 0.12 (0.09-0.17) | 0.11 (0.09-0.14) | 0.50 |
| C6OH (Hydroxyhexanoylcarnitine) | 0.04 (0.03-0.04) | 0.03 (0.02-0.04) | 0.16 |
| C6:1 (Hexenoylcarnitine) | 0.03 (0.02-0.06) | 0.02 (0.01-0.06) | 0.12 |
| C8:1 (Octenoylcarnitine) | 0.07 (0.04-0.14) | 0.05 (0.04-0.12) | 0.72 |
| C10:2 (Decadienoylcarnitine) | 0.04 (0.03-0.05) | 0.03 (0.02-0.05) | 0.25 |
| C12OH (Hydroxydodecanoylcarnitine) | 0.02 (0.02-0.03) | 0.02 (0.01-0.03) | 0.83 |
| C14OH (Hydroxytetradecanoylcarnitine) | 0.03 (0.02-0.03 | 0.02 (0.02-0.03) | 0.12 |
| C16:1OH (Hydroxyhexadecenoylcarnitine) | 0.04 (0.03-0.05) | 0.04 (0.03-0.04) | 0.43 |
| C18OH (Hydroxyoctadecanoylcarnitine) | 0.02 (0.01-0.02) | 0.02 (0.01-0.02) | 0.45 |
| C18:2 (Octadecadienylcarnitine) | 0.35 (0.22-0.50) | 0.26 (0.18-0.38) | 0.12 |

Variables are summarized as median (25^th^-75^th^ percentiles). BW is expressed in grams, GA in weeks, and all the other metabolites in µM.

**Supplementary Table S2. All the measured variables in penicillins+aminoglycoside-treated male and female EPI.**

| **Variables** | **Penicillins+aminoglycoside-treated**  **male EPI**  **(n=14)** | **Penicillins+aminoglycoside-treated**  **female EPI**  **(n=18)** | **p-value** |
| --- | --- | --- | --- |
| Ala | 128.2 (96.7-156.8) | 133.5 (92.7-185.4) | 0.44 |
| Val | 93.3 (80.6-132.4) | 92.0 (68.1-125.9) | 0.96 |
| Xle | 104.2 (76.9-120.1) | 99.8 (76.6-141.6) | 0.92 |
| Met | 15.4 (11.0-18.5) | 18.4 (9.8-30.8) | 0.48 |
| Phe | 46.3 (39.3-55.7) | 45.3 (41.1-57.0) | 0.69 |
| Tyr | 45.6 (33.6-90.4) | 65.3 (44.6-106.3) | 0.31 |
| Asp | 19.5 (16.2-22.0) | 19.8 (16.6-25.4) | 0.46 |
| Glu | 166.2 (139.1-214.2) | 176.7 (140.6-208.2) | 0.80 |
| Gly | 219.3 (180.1-301.4) | 225.5 (215.0-308.5) | 0.56 |
| Orn | 33.5 (29.8-43.0) | 37.2 (27.4-42.9) | 0.96 |
| Cit | 8.5 (7.1-10.4) | 10.7 (6.1-12.7) | 0.38 |
| Arg | 6.8 (6.1-8.1) | 8.1 (4.1-15.5) | 0.25 |
| C0 | 33.1 (23.8-40.4) | 20.6 (15.1-29.9) | 0.009 |
| C2 | 25.0 (18.6-34.4) | 17.6 (14.9-23.5) | 0.022 |
| C3 | 2.9 (2.0-3.4) | 2.1 (1.4-2.7) | 0.11 |
| C4 | 0.7 (0.4-0.6) | 0.4 (0.2-0.5) | 0.026 |
| C5:1 | 0.03 (0.02-0.04) | 0.02 (0.02-0.03) | 0.15 |
| C5 | 0.3 (0.2-0.5) | 0.3 (0.2-0.3) | 0.15 |
| C6 | 0.05 (0.04-0.08) | 0.03 (0.03-0.04) | 0.032 |
| C5OH | 0.1 (0.13-0.18) | 0.1 (0.11-0.16) | 0.51 |
| C8 | 0.1(0.06-0.13) | 0.09 (0.06-0.12) | 0.25 |
| C3DC | 0.05 (0.04-0.06) | 0.04 (0.03-0.05) | 0.025 |
| C10:1 | 0.07 (0.06-0.09) | 0.05 (0.04-0.08) | 0.091 |
| C10 | 0.07 (0.05-0.08) | 0.05 (0.04-0.07) | 0.17 |
| C4DC | 0.12 (0.09-0.14) | 0.11 (0.09-0.14) | 0.34 |
| C5DC | 0.06 (0.05-0.07) | 0.05 (0.03-0.06) | 0.11 |
| C12:1 | 0.04 (0.03-0.04) | 0.03 (0.02-0.04) | 0.08 |
| C12 | 0.09 (0.06-0.11) | 0.06 (0.06-0.08) | 0.05 |
| C6DC | 0.045 (0.03-0.08) | 0.05 (0.03-0.06) | 0.88 |
| C14:2 | 0.09 (0.07-0.12) | 0.07 (0.06-0.10) | 0.36 |
| C14:1 | 0.1 (0.1-0.2) | 0.1 (0.07-0.1) | 0.04 |
| C14 | 0.2 (0.1-0.2) | 0.1 (0.08-0.2) | 0.025 |
| C8DC | 0.03 (0.02-0.04) | 0.04 (0.03-0.04) | 0.59 |
| C16:1 | 0.2 (0.1-0.2) | 0.11 (0.08-0.2) | 0.026 |
| C16 | 1.5 (1.1-1.7) | 1.03 (0.7-1.2) | 0.02 |
| C10DC | 0.3 (0.2-0.3) | 0.2 (0.1-0.2) | 0.035 |
| C16OH | 0.03 (0.02-0.03) | 0.02 (0.016-0.025) | 0.02 |
| C18:1 | 1.7 (1.1-1.9) | 1.0 (0.7-1.5) | 0.007 |
| C18 | 0.8 (0.6-0.9) | 0.6 (0.5-0.7) | 0.044 |
| C18:1OH | 0.04 (0.03-0.06) | 0.03 (0.02-0.04) | 0.13 |
| C4OH | 0.13 (0.11-0.15) | 0.10 (0.07-0.14) | 0.048 |
| C6OH | 0.05 (0.03-0.05) | 0.04 (0.02-0.04) | 0.18 |
| C6:1 | 0.03 (0.02-0.07) | 0.02 (0.02-0.03) | 0.27 |
| C8:1 | 0.07 (0.06-0.13) | 0.07 (0.04-0.11) | 0.58 |
| C10:2 | 0.04 (0.03-0.06) | 0.04 (0.03-0.06) | 0.98 |
| C12OH | 0.03 (0.02-0.03) | 0.03 (0.02-0.03) | 0.60 |
| C14OH | 0.03 (0.02-0.04) | 0.02 (0.01-0.02) | 0.062 |
| C16:1OH | 0.05 (0.03-0.06) | 0.04 (0.03-0.04) | 0.019 |
| C18OH | 0.02 (0.01-0.02) | 0.02 (0.01-0.02) | 0.25 |
| C18:2 | 0.3 (0.2-0.5) | 0.37 (0.2-0.4) | 0.98 |

Variables are summarized as median (25^th^-75^th^ percentiles). BW is expressed in grams, GA in weeks, and all the other metabolites in µM.

**Supplementary Table S3. All the measured variables in antibiotics-treated VS. untreated groups for male and female EPI.**

| **Variables** | **Untreated**  **male EPI**  **(n=20)** | **Antibiotics-treated**  **male** **EPI**  **(n=37)** | **p-value** | **Untreated**  **female EPI**  **(n=22)** | **Antibiotics-treated**  **female EPI**  **(n=37)** | **p-value** | |  |
| --- | --- | --- | --- | --- | --- | --- | --- | --- |
| BW | 842.25 (168.70) | 810.62 (156.72) | 0.48 | 737.50 (655.00-820.00) | 810.00 (690.00-950.00) | | 0.31 | |
| GA | 26.00 (26.00-27.00) | 25.00 (25.00-27.00) | 0.21 | 25.00 (24.00-26.00) | 26.00 (25.00-27.00) | | 0.11 | |
| Ala | 135.044 (53.07) | 137.57 (55.62) | 0.87 | 139.04 (110.01-158.72) | 127.87 (91.96-152.87) | | 0.45 | |
| Val | 84.24 (69.40-129.31) | 93.63 (78.28-131.99) | 0.45 | 93.14 (79.41-106.61) | 92.33 (68.11-119.61) | | 0.76 | |
| Xle | 93.65 (67.62-116.63) | 104.39 (80.11-128.78) | 0.10 | 103.72 (72.08-124.12) | 99.61 (82.08-114.58) | | 0.87 | |
| Met | 14.50 (10.25- 21.52) | 18.32 (12.28-24.41) | 0.24 | 17.10 (11.31-27.49) | 17.31 (10.75-21.44) | | 0.70 | |
| Phe | 44.53 (38.02-51.82) | 49.04 (43.06-59.23) | 0.10 | 44.49 (39.60-72.68) | 44.39 (41.06-52.44) | | 0.42 | |
| Tyr | 42.29 (27.94-78.85) | 60.05 (39.82-90.43) | 0.08 | 45.21 (32.40-136.91) | 59.92 (32.24-69.93) | | 0.80 | |
| Asp | 21.62 (15.80-24.33) | 20.45 (15.30-28.69) | 0.95 | 20.75 (14.94-27.68) | 19.69 (16.58-25.50) | | 0.92 | |
| Glu | 166.35 (51.88) | 184.7335 (59.49) | 0.25 | 156.53 (127.93-197.84) | 180.74 (160.35-208.22) | | 0.12 | |
| Gly | 244.55 (61.56) | 262.59 (77.60) | 0.37 | 288.42 (229.03-304.13) | 227.86 (204.26-303.53) | | 0.12 | |
| Orn | 28.90 (21.16-36.26) | 39.58 (33.06-47.52) | 0.001 | 32.96 (21.32-54.07) | 37.19 (27.37-42.72) | | 0.60 | |
| Cit | 8.11 (5.64-9.87) | 10.29 (7.46-14.80) | 0.03 | 8.51 (6.47-11.85) | 8.52 (6.88-11.70) | | 0.88 | |
| Arg | 7.81 (4.69-9.82) | 8.47 (6.02-14.24) | 0.27 | 7.11 (2.91-10.71) | 7.20 (5.22-9.39) | | 0.37 | |
| C0 | 26.24 (17.80-38.74) | 35.49 (27.83-57.60) | 0.03 | 19.66 (17.10-31.49) | 24.89 (17.70-36.46) | | 0.54 | |
| C2 | 20.79 (16.49-28.43) | 25.91 (20.72-37.07) | 0.02 | 18.84 (14.67-20.45) | 20.88 (15.44-29.38) | | 0.18 | |
| C3 | 2.68 (1.79 -3.97) | 3.06 (2.12-3.92) | 0.34 | 1.95 (1.32-2.63) | 2.19 (1.51-3.17) | | 0.20 | |
| C4 | 0.40 (0.29-0.63) | 0.54 (0.44-0.89) | 0.01 | 0.41 (0.32-0.47) | 0.46 (0.34-0.53) | | 0.28 | |
| C5:1 | 0.02 (0.02-0.04) | 0.03 (0.02-0.04) | 0.15 | 0.03 (0.02-0.03) | 0.03 (0.02-0.04) | | 0.72 | |
| C5 | 0.28 (0.20-0.41) | 0.38 (0.29-0.70) | 0.02 | 0.32 (0.22-0.39) | 0.30 (0.24-0.38) | | 0.86 | |
| C6 | 0.05 (0.03-0.07) | 0.05 (0.04-0.08) | 0.36 | 0.04 (0.04-0.04) | 0.04 (0.03-0.05) | | 0.96 | |
| C5OH | 0.15 (0.12 -0.19) | 0.18 (0.15-0.21) | 0.20 | 0.15 (0.11-0.18) | 0.15 (0.11-0.18) | | 0.83 | |
| C8 | 0.09 (0.06-0.16) | 0.11 (0.08-0.14) | 0.45 | 0.07 (0.06-0.10) | 0.09 (0.08-0.13) | | 0.08 | |
| C3DC | 0.04 (0.03-0.05) | 0.05 (0.04-0.07) | 0.10 | 0.03 (0.03-0.05) | 0.04 (0.03-0.05) | | 0.15 | |
| C10:1 | 0.06 (0.05-0.09) | 0.08 (0.06-0.13) | 0.13 | 0.05 (0.04-0.09) | 0.05 (0.04-0.15) | | 0.31 | |
| C10 | 0.06 (0.04-0.10) | 0.07 (0.06-0.11) | 0.22 | 0.05 (0.04 -0.08) | 0.06 (0.04-0.09) | | 0.47 | |
| C4DC | 0.11 (0.08-0.17) | 0.12 (0.10-0.17) | 0.23 | 0.09 (0.08-0.16) | 0.10 (0.09-0.12) | | 0.47 | |
| C5DC | 0.06 (0.04-0.07) | 0.06 (0.04-0.08) | 0.36 | 0.05 (0.04-0.06) | 0.05 (0.04-0.07) | | 0.24 | |
| C12:1 | 0.03 (0.02-0.05) | 0.04 (0.03-0.06) | 0.35 | 0.03 (0.02-0.05) | 0.03 (0.03-0.08) | | 0.45 | |
| C12 | 0.08 (0.06-0.14) | 0.08 (0.06-0.12) | 0.712 | 0.07 (0.05-0.17) | 0.08 (0.06-0.11) | | 0.99 | |
| C6DC | 0.04 (0.03-0.07) | 0.04 (0.03-0.06) | 0.98 | 0.04 (0.03-0.06) | 0.05 (0.03-0.07) | | 0.27 | |
| C14:2 | 0.07 (0.06-0.09) | 0.08 (0.07-0.11) | 0.08 | 0.06 (0.04-0.08) | 0.07 (0.05-0.10) | | 0.12 | |
| C14:1 | 0.13 (0.09-0.17) | 0.15 (0.12-0.23) | 0.10 | 0.10 (0.07-0.14) | 0.12 (0.08-0.17) | | 0.20 | |
| C14 | 0.16 (0.12-0.23) | 0.17 (0.12-0.21) | 0.92 | 0.13 (0.10-0.18) | 0.14 (0.11-0.20) | | 0.67 | |
| C8DC | 0.04 (0.02-0.04) | 0.03 (0.02-0.04) | 0.96 | 0.03 (0.02-0.04) | 0.04 (0.03-0.04) | | 0.12 | |
| C16:1 | 0.12 (0.09-0.16) | 0.17 (0.12-0.21) | 0.02 | 0.10 (0.09-0.16) | 0.12 (0.08-0.19) | | 0.53 | |
| C16 | 1.07 (0.79-1.55) | 1.14 (0.98-1.76) | 0.62 | 1.00 (0.64-1.48) | 1.07 (0.75-1.37) | | 0.94 | |
| C10DC | 0.25 (0.18-0.38) | 0.24 (0.18-0.40) | 0.99 | 0.33 (0.16-0.56) | 0.20 (0.10-0.33) | | 0.03 | |
| C16OH | 0.02 (0.02-0.03) | 0.03 (0.02-0.03) | 0.09 | 0.02 (0.01-0.03) | 0.02 (0.02-0.03) | | 0.11 | |
| C18:1 | 1.09 (0.40) | 1.54 (0.65) | 0.01 | 0.94 (0.56 -1.30) | 1.04 (0.75-1.47) | | 0.48 | |
| C18 | 0.66 (0.46-0.88) | 0.71 (0.59-0.92) | 0.45 | 0.62 (0.44-0.81) | 0.59 (0.50-0.77) | | 0.89 | |
| C18:1OH | 0.03 (0.02-0.03) | 0.04 (0.02-0.05) | 0.26 | 0.02 (0.02-0.03) | 0.03 (0.02-0.04) | | 0.05 | |
| C4OH | 0.12 (0.09- 0.17) | 0.15 (0.11-0.22) | 0.11 | 0.11 (0.09-0.14) | 0.13 (0.08-0.18) | | 0.43 | |
| C6OH | 0.04 (0.03-0.04) | 0.04 (0.03-0.05) | 0.25 | 0.03 (0.02 -0.04) | 0.04 (0.03-0.04) | | 0.24 | |
| C6:1 | 0.03 (0.02-0.06) | 0.03 (0.02-0.05) | 0.44 | 0.02 (0.01-0.06) | 0.03 (0.02-0.03) | | 0.79 | |
| C8:1 | 0.07 (0.04-0.14) | 0.09 (0.06-0.13) | 0.73 | 0.05 (0.04-0.12) | 0.06 (0.04-0.14) | | 0.62 | |
| C10:2 | 0.04 (0.03-0.05) | 0.04 (0.03-0.06) | 0.35 | 0.03 (0.02-0.05) | 0.04 (0.02-0.06) | | 0.18 | |
| C12OH | 0.02 (0.02-0.03) | 0.03 (0.02-0.03) | 0.05 | 0.02 (0.01-0.03) | 0.03 (0.02-0.04) | | 0.11 | |
| C14OH | 0.03 (0.02-0.03) | 0.03 (0.03-0.04) | 0.39 | 0.02 (0.02-0.03) | 0.03 (0.02-0.04) | | 0.19 | |
| C16:1OH | 0.04 (0.03-0.05) | 0.05 (0.03-0.06) | 0.27 | 0.04 (0.03-0.04) | 0.04 (0.03-0.05) | | 0.35 | |
| C18OH | 0.02 (0.01-0.02) | 0.02 (0.01-0.02) | 0.39 | 0.02 (0.01-0.02) | 0.02 (0.01-0.02) | | 0.10 | |
| C18:2 | 0.35 (0.22-0.50) | 0.37 (0.26-0.47) | 0.89 | 0.26 (0.18-0.38) | 0.32 (0.19-0.45) | | 0.34 | |

Variables are summarized as mean (SD) or median (25^th^-75^th^ percentiles). BW is expressed in grams, GA in weeks, and all the other metabolites in µM.

**Supplementary Tables S4. Correlation coefficients between BW and study variables.**

| **Variables**  **Rho (p-value)** | **Relationship between BW and study variables** | | | |
| --- | --- | --- | --- | --- |
|  | **Untreated males** | **Untreated females** | **Antibiotics males** | **Antibiotics females** |
| GA | 0.34 (0.15) | 0.33 (0.14) | 0.50 (0.002) | 0.68 (<0.0001) |
| Ala | -0.44 (0.05) | -0.08 (0.71) | -0.21 (0.21) | -0.11 (0.53) |
| Val | -0.36 (0.12) | -0.19 (0.40) | -0.14 (0.40) | -0.10 (0.55) |
| Xle | -0.26 (0.27) | -0.18 (0.42) | 0.02 (0.90) | -0.07 (0.69) |
| Met | -0.13 (0.60) | -0.15 (0.52) | 0.04 (0.84) | 0.07 (0.70) |
| Phe | -0.19 (0.42) | -0.15 (0.52) | -0.08 (0.65) | -0.01 (0.95) |
| Tyr | 0.11 (0.63) | -0.21 (0.35) | 0.02 (0.90) | -0.10 (0.56) |
| Asp | -0.04 (0.86) | -0.19 (0.41) | -0.07 (0.67) | 0.10 (0.55) |
| Glu | 0.13 (0.59) | 0.22 (0.33) | -0.23 (0.17) | 0.02 (0.92) |
| Gly | -0.32 (0.18) | -0.07 (0.77) | -0.10 (0.54) | 0.09 (0.60) |
| Orn | -0.48 (0.03) | -0.24 (0.28) | -0.16 (0.35) | 0.20 (0.24) |
| Cit | -0.47 (0.04) | -0.20 (0.38) | -0.01 (0.95) | -0.20 (0.24) |
| Arg | -0.35 (0.14) | -0.23 (0.30) | 0.02 (0.93) | -0.05 (0.98) |
| C0 | 0.10 (0.67) | 0.13 (0.55) | -0.26 (0.12) | 0.08 (0.65) |
| C2 | 0.23 (0.33) | 0.20 (0.37) | -0.20 (0.23) | 0.16 (0.34) |
| C3 | 0.18 (0.46) | 0.14 (0.53) | -0.20 (0.23) | 0.25 (0.14) |
| C4 | 0.05 (0.85) | 0.06 (0.81) | -0.32 (0.05) | 0.10 (0.54) |
| C5:1 | 0.10 (0.67) | -0.20 (0.36) | 0.14 (0.42) | -0.17 (0.32) |
| C5 | -0.10 (0.67) | -0.34 (0.13) | -0.18 (0.29) | -0.07 (0.68) |
| C6 | -0.17 (0.48) | 0.29 (0.19) | -0.28 (0.09) | 0.21 (0.21) |
| C5OH | 0.22 (0.35) | -0.31 (0.16) | 0.01 (0.99) | -0.23 (0.16) |
| C8 | -0.36 (0.13) | -0.12 (0.59) | -0.17 (0.32) | -0.27 (0.11) |
| C3DC | -0.15 (0.53) | 0.13 (0.57) | -0.03 (0.89) | -0.07 (0.69) |
| C10:1 | -0.23 (0.32) | -0.26 (0.25) | -0.08 (0.96) | -0.03 (0.85) |
| C10 | -0.07 (0.76) | 0.02 (0.92) | -0.11 (0.52) | 0.06 (0.74) |
| C4DC | 0.31 (0.19) | -0.06 (0.78) | -0.12 (0.47) | 0.03 (0.86) |
| C5DC | -0.18 (0.44) | -0.15 (0.50) | -0.01 (0.98) | -0.11 (0.52) |
| C12:1 | -0.02 (0.93) | -0.11 (0.62) | -0.17 (0.32) | 0.12 (0.49) |
| C12 | 0.09 (0.69) | 0.15 (0.52) | -0.17 (0.31) | 0.18 (0.30) |
| C6DC | 0.21 (0.37) | -0.05 (0.84) | 0.06 (0.72) | 0.13 (0.45) |
| C14:2 | -0.20 (0.39) | -0.11 (0.63) | -0.14 (0.42) | -0.11 (0.51) |
| C14:1 | -0.04 (0.88) | -0.001 (1.00) | -0.24 (0.16) | 0.18 (0.28) |
| C14 | -0.03 (0.92) | 0.22 (0.33) | -0.27 (0.10) | 0.37 (0.02) |
| C8DC | -0.32 (0.17) | -0.04 (0.86) | -0.06 (0.97) | -0.09 (0.59) |
| C16:1 | -0.15 (0.54) | 0.21 (0.34) | -0.29 (0.08) | 0.34 (0.04) |
| C16 | 0.18 (0.44) | 0.15 (0.51) | -0.34 (0.04) | 0.39 (0.02) |
| C10DC | 0.32 (0.17) | 0.08 (0.71) | 0.11 (0.50) | 0.15 (0.38) |
| C16OH | 0.01 (0.96) | -0.18 (0.43) | -0.19 (0.26) | 0.01 (1.00) |
| C18:1 | 0.09 (0.72) | 0.03 (0.90) | -0.35 (0.03) | 0.13 (0.45) |
| C18 | 0.06 (0.80) | 0.02 (0.92) | -0.19 (0.25) | 0.24 (0.15) |
| C18:1OH | 0.01 (0.96) | 0.13 (0.56) | -0.27 (0.10) | -0.20 (0.24) |
| C4OH | 0.28 (0.23) | -0.22 (0.32) | -0.06 (0.75) | 0.18 (0.28) |
| C6OH | 0.05 (0.83) | 0.33 (0.13) | -0.23 (0.17) | 0.02 (0.90) |
| C6:1 | -0.05 (0.83) | -0.10 (0.66) | 0.11 (0.54) | -0.38 (0.02) |
| C8:1 | -0.42 (0.06) | -0.22 (0.33) | 0.17 (0.33) | -0.03 (0.87) |
| C10:2 | -0.45 (0.05) | 0.15 (0.51) | 0.11 (0.52) | 0.01 (0.93) |
| C12OH | -0.36 (0.12) | -0.16 (0.48) | 0.08 (0.63) | -0.09 (0.61) |
| C14OH | -0.07 (0.78) | 0.18 (0.43) | -0.23 (0.17) | -0.03 (0.86) |
| C16:1OH | 0.01 (0.99) | 0.05 (0.84) | -0.24 (0.16) | -0.07 (0.67) |
| C18OH | 0.01 (0.98) | -0.10 (0.67) | -0.19 (0.26) | -0.07 (0.67) |
| C18:2 | -0.01 (0.99) | -0.08 (0.72) | 0.22 (0.19) | 0.16 (0.36) |

Data are reported as Pearson correlation coefficients, with p-values indicated in brackets

**Supplementary Tables S5. Correlation coefficients between GA and study variables.**

| **Variables**  **Rho (p-value)** | **Relationship between GA and study variables** | | | |
| --- | --- | --- | --- | --- |
|  | **Untreated EPI** | **Untreated EPI** | **Antibiotic treated male EPI** | **Antibiotic treated female EPI** |
| BW | 0.38 (0.10) | 0.40 (0.07) | 0.50 (0.02) | 0.68 (<0.0001) |
| Ala | -0.39 (0.09) | 0.46 (0.03) | -0.13 (0.43) | -0.13 (0.44) |
| Val | -0.27 (0.24) | 0.27 (0.23) | -0.04 (0.81) | -0.24 (0.15) |
| Xle | -0.27 (0.26) | 0.27 (0.23) | 0.09 (0.61) | -0.34 (0.04) |
| Met | 0.24 (0.31) | 0.24 (0.29) | 0.10 (0.57) | -0.25 (0.13) |
| Phe | -0.29 (0.21) | 0.28 (0.21) | 0.06 (0.73) | -0.28 (0.10) |
| Tyr | -0.16 (0.51) | -0.03 (0.91) | 0.01 (0.99) | -0.33 (0.04) |
| Asp | -0.41 (0.07) | 0.48 (0.02) | -0.27 (0.10) | -0.14 (0.40) |
| Glu | -0.45 (0.045) | 0.26 (0.24) | -0.27 (0.11) | -0.19 (0.27) |
| Gly | -0.46 (0.04) | 0.40 (0.07) | -0.09 (0.61) | -0.08 (0.62) |
| Orn | 0.01 (0.97) | 0.38 (0.08) | -0.06 (0.71) | -0.08 (0.62) |
| Cit | -0.16 (0.52) | 0.06 (0.79) | -0.11 (0.52) | -0.31 (0.06) |
| Arg | 0.20 (0.39) | 0.18 (0.43) | -0.14 (0.41) | -0.25 (0.14) |
| C0 | -0.35 (0.13) | 0.28 (0.21) | -0.05 (0.75) | 0.32 (0.06) |
| C2 | -0.27 (0.25) | 0.15 (0.50) | -0.06 (0.74) | 0.20 (0.25) |
| C3 | -0.51 (0.02) | -0.01 (0.98) | -0.23 (0.17) | 0.20 (0.25) |
| C4 | -0.55 (0.01) | 0.30 (0.17) | -0.26 (0.12) | 0.24 (0.15) |
| C5:1 | -0.20 (0.39) | 0.15 (0.51) | 0.09 (0.58) | -0.02 (0.89) |
| C5 | -0.36 (0.12) | 0.11 (0.63) | -0.16 (0.35) | -0.02 (0.90) |
| C6 | -0.12 (0.62) | 0.29 (0.18) | -0.20 (0.23) | 0.20 (0.24) |
| C5OH | -0.44 (0.05) | -0.25 (0.25) | -0.15 (0.39) | -0.28 (0.09) |
| C8 | -0.22 (0.35) | 0.32 (0.15) | -0.15 (0.39) | -0.06 (0.71) |
| C3DC | -0.18 (0.45) | 0.13 (0.58) | -0.04 (0.80) | 0.06 (0.72) |
| C10:1 | 0.07 (0.78) | 0.11 (0.61) | 0.07 (0.68) | -0.01 (0.99) |
| C10 | 0.01 (0.98) | 0.20 (0.37) | -0.01 (0.96) | 0.25 (0.13) |
| C4DC | -0.02 (0.95) | 0.04 (0.85) | -0.02 (0.92) | 0.15 (0.36) |
| C5DC | 0.07 (0.76) | 0.06 (0.80) | 0.01 (0.96) | 0.12 (0.49) |
| C12:1 | -0.13 (0.60) | -0.08 (0.72) | -0.05 (0.76) | 0.21 (0.22) |
| C12 | -0.18 (0.46) | 0.05 (0.83) | -0.23 (0.16) | 0.34 (0.04) |
| C6DC | 0.04 (0.87) | 0.01 (0.98) | -0.08 (0.66) | -0.02 (0.89) |
| C14:2 | -0.11 (0.65) | 0.17 (0.45) | -0.15 (0.38) | -0.17 (0.32) |
| C14:1 | -0.25 (0.28) | 0.04 (0.86) | -0.37 (0.03) | 0.28 (0.09) |
| C14 | -0.43 (0.06) | -0.09 (0.69) | -0.16 (0.35) | 0.43 (0.01) |
| C8DC | -0.11 (0.65) | 0.20 (0.38) | -0.12 (0.48) | 0.12 (0.49) |
| C16:1 | -0.48 (0.03) | -0.15 (0.50) | -0.20 (0.23) | 0.45 (0.01) |
| C16 | -0.23 (0.32) | -0.03 (0.91) | -0.06 (0.71) | 0.51 (0.001) |
| C10DC | -0.18 (0.44) | -0.38 (0.08) | 0.17 (0.33) | 0.28 (0.10) |
| C16OH | -0.12 (0.61) | -0.22 (0.33) | -0.19 (0.26) | 0.14 (0.40) |
| C18:1 | -0.33 (0.16) | -0.07 (0.75) | -0.24 (0.16) | 0.31 (0.06) |
| C18 | -0.42 (0.07) | -0.23 (0.30) | -0.11 (0.52) | 0.35 (0.03) |
| C18:1OH | -0.08 (0.73) | 0.15 (0.51) | -0.33 (0.05) | -0.11 (0.53) |
| C4OH | -0.11 (0.64) | 0.01 (0.97) | -0.26 (0.13) | 0.18 (0.28) |
| C6OH | -0.43 (0.06) | 0.27 (0.23) | -0.17 (0.31) | 0.01 (0.98) |
| C6:1 | 0.12 (0.61) | 0.26 (0.24) | 0.16 (0.35) | -0.25 (0.13) |
| C8:1 | -0.04 (0.85) | 0.18 (0.43) | 0.05 (0.75) | -0.10 (0.56) |
| C10:2 | -0.17 (0.48) | 0.20 (0.38) | 0.06 (0.73) | -0.10 (0.54) |
| C12OH | -0.08 (0.73) | 0.03 (0.90) | -0.18 (0.30) | 0.17 (0.32) |
| C14OH | -0.02 (0.93) | 0.07 (0.75) | -0.23 (0.17) | 0.05 (0.75) |
| C161OH | -0.18 (0.44) | 0.02 (0.91) | -0.20 (0.23) | 0.17 (0.31) |
| C18OH | 0.02 (0.92) | -0.16 (0.48) | -0.19 (0.25) | -0.12 (0.49) |
| C18:2 | 0.01 (1.00) | 0.01 (0.99) | -0.06 (0.73) | 0.18 (0.30) |

Data are reported as Pearson correlation coefficients, with p-values indicated in brackets
